# Effect of the Combination of Gold Nanoparticles and Polyelectrolyte Layers on SERS Measurements

**DOI:** 10.3390/bios12100895

**Published:** 2022-10-19

**Authors:** Antonello Nucera, Rossella Grillo, Carmen Rizzuto, Riccardo Cristoforo Barberi, Marco Castriota, Thomas Bürgi, Roberto Caputo, Giovanna Palermo

**Affiliations:** 1 Department of Physics, University of Calabria, Via Ponte Bucci, Cubo 31C, 87036 Rende, Cosenza, Italy; 2 CNR-Nanotec, Via Ponte Bucci, Cubo 33C, 87036 Rende, Cosenza, Italy; 3 Department of Physical Chemistry, University of Geneva, 30 Quai Ernest-Ansermet, 1211 Geneva, Switzerland

**Keywords:** SERS, gold nanoparticles, polyelectrolyte layers, plasmonic band, Raman spectroscopy

## Abstract

In this study, polyelectrolyte (PE) layers are deposited on substrates made by glass covered with an array of gold nanoparticles (GNPs). In particular, the samples studied have 0 PE layers (GGPE${}_{0}$), 3 PE layers (GGPE${}_{3}$), 11 PE layers (GGPE${}_{11}$), and 21 PE layers (GGPE${}_{21}$). All samples have been studied by micro-Raman spectroscopy. An acetic acid solution (10% *v*/*v*) has been used as a standard solution in order to investigate the SERS effect induced by different numbers of PE layers in each sample. The Surface Enhancement Raman Spectroscopy (SERS) effect correlating to the number of PE layers deposited on the samples has been shown. This effect is explained in terms of an increase in the interaction between the photon of the laser source and the plasmonic band of the GNPs due to a change of the permittivity of the surrounding medium around the GNPs. The trends of the ratios of the intensities of the Raman bands of the acetic acid solution (acetic acid and water molecules) on the band at 1098 cm${}^{-1}$ ascribed to the substrates increase, and the number of PE layers increases.

## 1. Introduction

Raman spectroscopy, in the last decades, has shown to be a very useful tool for studying many different types of materials, such as thin films [1], gels [2], carbon-based materials [3,4], layered semiconductors [5], polymers [6], materials of biological [7] interest, and also for cultural heritage [8] issue. Sometimes, when Raman features are very low in intensity or are not detectable at all, it is possible to make the analyte contact a particular surface that is usually made up of a noble metal, which is able to enhance Raman signals by using an effect due to the interaction between the light and the plasmonic surface on the analyte [9]. Such a technique is called Surface Enhancement Raman Spectroscopy (SERS) [9]. SERS is currently well-known and widely used in various studies, such as the following: the detection of low abundance molecules [10,11], monitoring environmental contaminants [12,13], food science [14], analyses of biomolecules, pharmaceutical and drugs research, and many others [15,16]. SERS was observed for the first time in 1974 [17,18], and it is a technique that allows the enhancement of signals detected by normal Raman spectroscopy. Briefly, SERS occurs due to electronic and chemical interactions between the sample, the substrate, and the laser source. To explain the SERS effect, the mechanisms involved are the following two: the electromagnetic and charge transfer. The first one occurs when the incident laser source excites the surface plasmons of the metal substrate, and the second one is ascribed to a transfer of electrons between the metal and sample [12]. Noble metal surfaces have been used as SERS substrates. Those have been used as flat surfaces or as nanoparticles [19]. Today, there are several methods for obtaining SERS substratess: metal nanoparticles in suspension, metal nanoparticles immobilized on solid substrates, and nanostructures fabricated directly on solid substrates [20,21,22]. Usually, the substrates used to produce SERS are obtained with Electronic Beam Lithography (EBL), but it is well known that the costs for the realization of these types of substrates are quite high. For this reason, it is necessary to find alternative manufacturing methods that can lead to the manufacture of large substrates, such as a microscope slide, in a quick and low-cost manenr. The SERS analysis can make a strong contribution to the development of high-performance sensing platforms in terms of sensitivity and selectivity when used in the early diagnosis of diseases and in the quick identification of bacteria and viruses [23,24]. Furthermore, in the use of biological materials, it is sometimes necessary that there is no direct contact between the layer of plasmonic material and the biological material in order to prevent a modification of the structure of biomolecules (e.g., peptides), due to their interactions with the plasmonic substrate. For this reason, it is important to study the use of new layers that do not affect the SERS response but, on the contrary, can enhance it [25,26]. In this paper, SERS substrates have been made by a combination of a single array of gold nanoparticles (GNPs) and different layers, ranging from 3 to 21, of polyelectrolyte (PE) materials. The effect of the number of PE layers on the SERS effect has been studied by using a solution of 10% acetic acid as a testing system.

## 2. Materials and Methods

**Figure 1:**
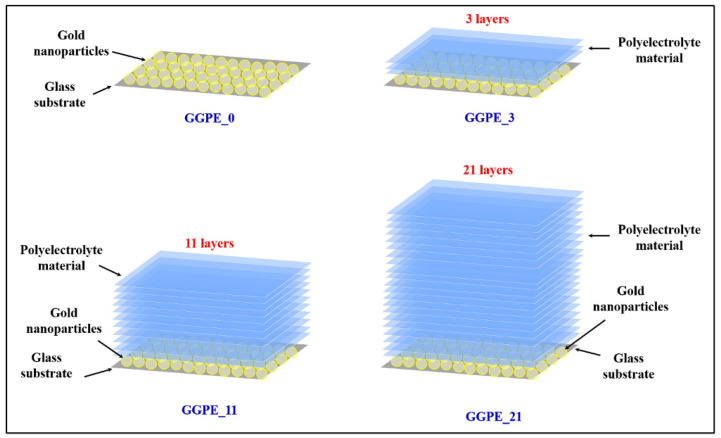
Schematic view of the four samples: GGPE${}_{0}$ (glass substrate/GNPs), GGPE${}_{3}$ (glass substrate/GNPs/3 layers of PE material), GGPE${}_{11}$ (glass substrate/GNPs/11 layers of PE material), and GGPE${}_{21}$ (glass substrate/GNPs/21 layer of PE material).

The substrates studied in this work are four samples composed of a GNP array on glass substrates covered by the following: 0 PE layers, 3 PE layers, 11 PE layers, and 21 PE layers labelled as GGPE${}_{0}$, GGPE${}_{3}$, GGPE${}_{11}$, and GGPE${}_{21}$, respectively. The PE layers are characterized by a thickness comparable with the size of the molecule (≈1 nm); for this reason, the thickness of the PE layers can be approximately 3 nm for GGPE${}_{3}$, 11 nm for the GGPE${}_{11}$, and 21 nm for the GGPE${}_{21}$. In Figure 1, a schematic view of the four sample studies is provided. The synthesis of GNPs, the functionalization of glass substrates, and the GNP’s array deposition have already been reported in our previous work [27]. Firstly, spherical GNPs have been synthesized following the Turkevich method [28]. In brief, 600 mL of an aqueous solution of tetrachloroauric (III) acid (0.25 mM) was brought to a boiling point in a round-bottomed flask under vigorous magnetic stirring. Subsequently, gold was reduced by pouring 15 mL of aqueous sodium citrate solution (0.03 M) into the boiling solution. After 15 min, the full reduction of the gold salt into monodisperse GNPs with an average diameter of 20 nm was obtained, as demonstrated by the solution color that turned from yellow to a deep red color. The glass substrates were first cleaned and hydroxylated with a piranha solution (3:1 mixture of sulphuric acid to hydrogen peroxide 30%) for 30 min. To modify the surface, the substrates were dipped in a 5% (*v*/*v*) solution of N-[3-(trimethoxysilyl)propyl] ethylenediamine in ethanol for 30 min and then rinsed with milli-Q water. Excess water was removed using a stream of nitrogen followed by drying in a furnace at 120 ${}^{{}^{\circ}}$C for 30 min to assure good silanization.

The GNP arrays were prepared by dipping the functionalized glass slides in the GNP solution for two and a half hours. The samples were then washed with milli-Q water and dried under a stream of nitrogen. The PE solutions of 5 mg/mL Poly(allylamine hydrochloride) (PAH, positively charged) and Poly(styrenesulfonate) (PSS, negatively charged) were prepared in 0.1 M of NaCl in water. Their opposite charge allows the fabrication of multilayers of PE layers via the layer-by-layer (LbL) deposition technique [29] by means of electrostatic interactions. For each configuration, we prepared and analyzed three samples of the same batch. The extinction cross section ($\sigma_{ext}$) of the samples, in the range 400–800 nm, is measured by means of a spectrophotometer (Cary 5000, by Agilent, Santa Clara, CA, United States).

**Figure 2:**
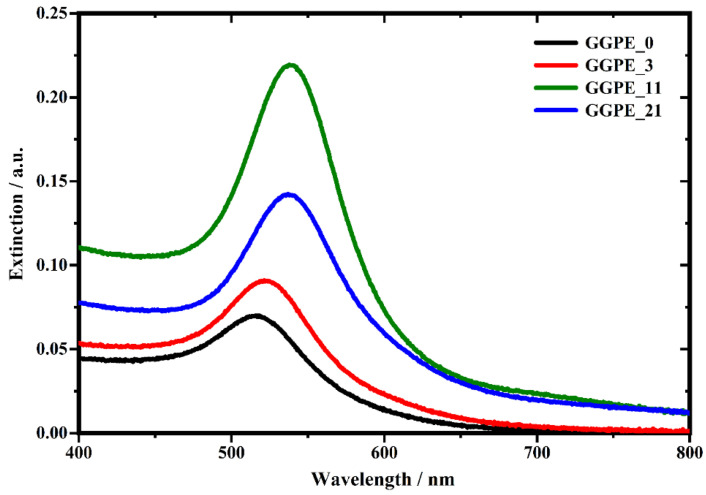
Localized surface plasmon band (LSPR) of the GGPE${}_{0}$, GGPE${}_{3}$, GGPE${}_{11}$, and GGPE${}_{21}$ sample.

The GNPs are characterized by a diameter of about 20 nm. For this size, scattering can be negligible and extinction can be considered equal to the absorbance of nano-objects. As we can see in the spectral response of the samples (Figure 2), the typical plasmonic band in the green region of the spectrum can be appreciated. In particular, the wavelength corresponding to the maximum of the plasmonic band ($\lambda_{LSPR}$) is at 514 nm for the GGPE${}_{0}$, at 522 nm for the GGPE${}_{3}$, 538 nm for the GGPE${}_{11}$, and 538 nm for the GGPE${}_{21}$. The absorption peak of the GNPs monolayer is significantly red-shifted by increasing the number of PE layers. This is due to the change of the refractive index surrounding the GNPs; in general, when metallic nanoparticles are immersed in a large refractive index medium, the resonance is shifted towards longer wavelengths [30]. Raman analyses were performed with a confocal Micro-Raman-LABRAM by Jobin Yvon Srl (now Horiba Scientific) with an objective long working distance 50× and equipped with Nd-YAG laser with a wavelength of 532 nm and a power of 50 mW. The detector is a CCD device cooled with a Peltier module (1024 × 256, 16 bits dynamic range (pixel size 27 μm)). All spectra have been collected by using a filter of OD 0.3 to decrease the power on the samples. The spectral resolution is about 1 cm${}^{-1}$. Acetic acid (99.0%, purity) was purchased by the Fluka Analytical Company. Such a solution was diluted at the 10% by using double-distilled water in order to be used as a standard for the Raman analysis. A drop of 10% *v*/*v* acetic acid solution was deposited on the samples, and then the Raman spectra were collected under the same operative conditions in order to compare the spectra coming from the different samples.

## 3. Results and Discussion

**Figure 3:**
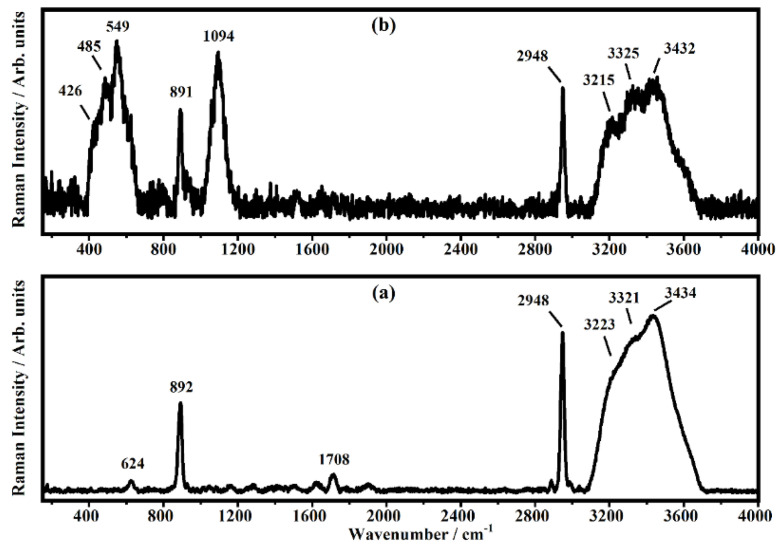
RepresentativeRaman spectra collected on the 10% *v*/*v* acetic acid solution (**a**) in a cuvette and (**b**) on the top surfaces of the glass substrate.

Acetic acid has been chosen as test substance because its vibrational modes in liquid and gaseous forms are well-known and generally precisely assigned in the literature [31,32,33,34]. In Figure 3, the representative Raman spectra collected on the 10% *v*/*v* acetic acid solution in a cuvette and on the top surfaces of the glass substrate that are used as references are shown.

**Figure 4:**
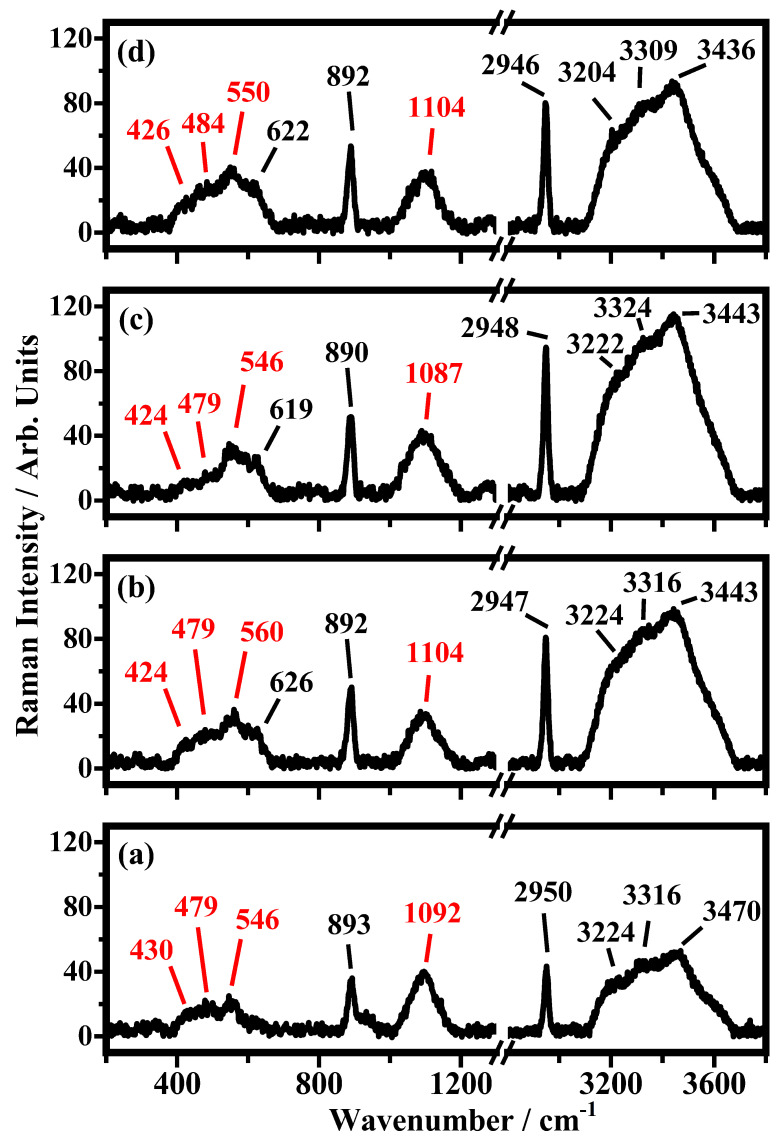
Representative Raman spectra of 10% *v*/*v* acetic acid solution in the ranges between 200 and 1300 cm${}^{-1}$ and between 2800 and 3800 cm${}^{-1}$ on (**a**) GGPE${}_{0}$ (glass substrate/GNPs), (**b**) GGPE${}_{3}$ (glass substrate/GNPs/3 layers of PE material), (**c**) GGPE${}_{11}$ (glass substrate/GNPs/11 layers of PE material), and (**d**) GGPE${}_{21}$ (glass substrate/GNPs/21 layer of PE material). The modes of the substrates are in red, while the modes of the solutions are in black. The peaks have been identified by comparisons with other spectra in the literature.

**Figure 5:**
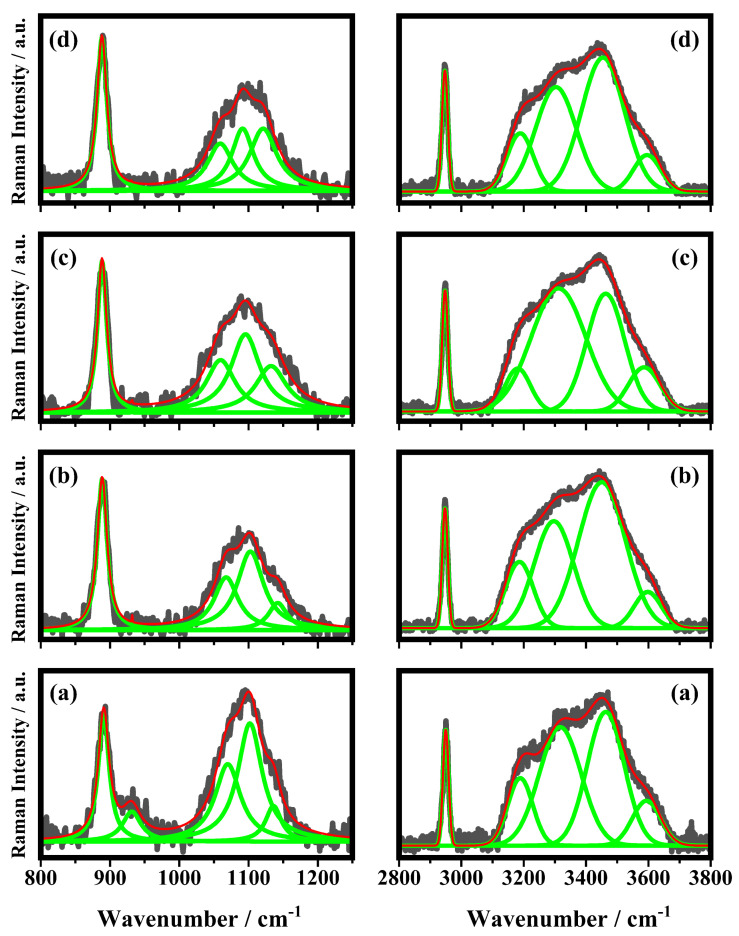
Fitting procedure in ranges between 800 and 1250 cm${}^{-1}$ (on the left) and between 2800 and 3800 cm${}^{-1}$ (on the right) of the Raman bands of 10% *v*/*v* acetic acid solution deposited on (**a**) GGPE${}_{0}$ (glass substrate/GNPs), (**b**) GGPE${}_{3}$ (glass substrate/GNPs/3 layers of PE material), (**c**) GGPE${}_{11}$ (glass substrate/GNPs/11 layers of PE material), and (**d**) GGPE${}_{21}$ (glass substrate/GNPs/21 layer of PE material). The grey line is the experimental spectra, the green lines are single fitting curves, and the red line is the total fitting curve.

The spectrum shown in Figure 3a is due to the volume of acetic acid: the entire laser spot is inside the liquid and the resulting Raman bands are very strong and well-defined. The three bands at 892 cm${}^{-1}$, 2948 cm${}^{-1}$, and the broad band at 3434 cm${}^{-1}$ have been assigned to the C-C stretching and C-H stretching of acetic acid molecules and to the O-H stretching of water molecules, respectively [31,32]. The bands at 3223 cm${}^{-1}$ and 3321 cm${}^{-1}$ have been assigned to O-H stretching modes [31,32]. The other two weaker bands at 624 cm${}^{-1}$ and 1708 cm${}^{-1}$ are attributed to the O=C-O bending mode and C=O stretching, respectively [31,32]. The representative Raman spectrum collected on acetic acid deposited on the top of the glass substrate is shown in Figure 3b. Besides the Raman bands described above (Figure 3a), which are still detected, there are new bands at 426 cm${}^{-1}$, 485 cm${}^{-1}$, 549 cm${}^{-1}$, and 1094 cm${}^{-1}$ that have been ascribed to the glass substrate [35]. The bands at 624 cm${}^{-1}$ and 1708 cm${}^{-1}$ seen in the spectrum of Figure 3a are not detected in the spectrum of Figure 3b. In Figure 4, the Raman spectra of the 10% *v*/*v* acetic acid solution deposited on the substrates with different numbers of PE are shown (see Figure 1). In the spectra of Figure 4, the bands that fall at 424 cm${}^{-1}$, 430 cm${}^{-1}$, 479 cm${}^{-1}$, 484 cm${}^{-1}$, 546 cm${}^{-1}$, 550 cm${}^{-1}$, 560 cm${}^{-1}$, 1087 cm${}^{-1}$, 1092 cm${}^{-1}$, and 1104 cm${}^{-1}$ are shown, and these are ascribed to the substrate; the bands at 619 cm${}^{-1}$, 622 cm${}^{-1}$, 626 cm${}^{-1}$, 890 cm${}^{-1}$, 892 cm${}^{-1}$, 893 cm${}^{-1}$, 2946 cm${}^{-1}$, 2947 cm${}^{-1}$, 2948 cm${}^{-1}$, and 2950 cm${}^{-1}$ are ascribed to acetic molecules, and the others bands above the 3000 cm${}^{-1}$ are due to the O-H stretching of water molecules. In Figure 5, some of the Raman bands of the spectra of Figure 4 are shown and fitted in order to study what happened to the samples when acetic acid is deposited on different substrates used in this study, and the number of PE layer changes is shown in Figure 1. It is possible to see in Figure 5 that the first band at 890 cm${}^{-1}$, ascribed to the acetic acid, is fitted by a single Lorentzian function (a second small band around 930 cm${}^{-1}$ is present in the sample without PE but becomes undetectable; in addition, the PE layer is deposited on the substrate). The other experimental band around 1100 cm${}^{-1}$ has been fitted by three functions that fall at about: 1065 cm${}^{-1}$, 1098 cm${}^{-1}$ and 1130 cm${}^{-1}$. The band in the middle seems to be the most representative Raman feature of the substrates, and it should be used as reference in order to monitor the effect of PE layers on the scattering response of the sample. The other Raman band at about 2950 cm${}^{-1}$, ascribed to acetic acid, has been fitted with just one single fitting function.

**Figure 6:**
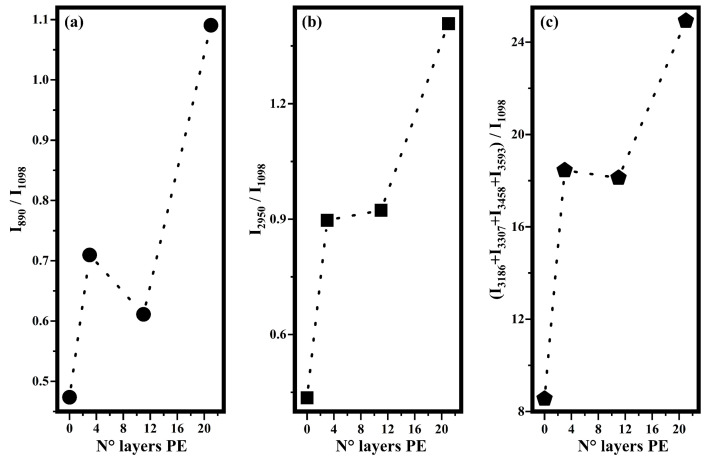
Trends, as a function of the number of the PE layers, of the ratios of intensities bands that fall at frequencies indicated as subscripts in I${}_{890}$/I${}_{1098}$ (**a**), I${}_{2950}$/I${}_{1098}$ (**b**), and (I${}_{3186}$ + I${}_{3307}$ + I${}_{3458}$ + I${}_{3593}$)/I${}_{1098}$ (**c**) and that have been calculated by the fitting procedure shown above (see Figure 4).

The other band, ascribed to the O-H stretching in water, has been fitted with four functions that fall at 3186 cm${}^{-1}$, 3307 cm${}^{-1}$, 3458 cm${}^{-1}$, and 3593 cm${}^{-1}$ in accordance with a previous study that has shown that the broad O-H band due to the water molecules was fitted with four Gaussian functions [34]. The first two bands, according to the literature [33,34], are ascribable to water molecules strongly associated with hydrogen bonds. The other band at 3458 cm${}^{-1}$ is ascribed to the presence of water molecules that are weakly associated (only one hydrogen atom involved in the hydrogen bond) and the last band at 3593 cm${}^{-1}$ is ascribed to completely free water molecules. In order to evaluate the effects on Raman features of the acetic acid solution performed by increasing the number of the PE layers, the intensities of the bands ascribed to acetic acid and to water on the intensity of the band at 1098 cm${}^{-1}$ have been plotted as a function of the layer of PE. In particular, in Figure 6, the ratios I${}_{890}$/I${}_{1098}$ (a), I${}_{2950}$/I${}_{1098}$ (b), and (I${}_{3186}$ + I${}_{3307}$ + I${}_{3458}$ + I${}_{3593}$)/I${}_{1098}$ (c) are shown. The trends shown in Figure 6 indicate that when the acetic acid solution is deposited on substrates with a higher number of PE layers, Raman scattering results are stronger then when it is deposited on substrates without any polyelectrolyte layers. In particular, in the first two panels of Figure 6a,b, it has been shown that the intensities of the bands ascribed to acetic acid molecules increased when they were deposited on sample GGPE${}_{21}$ with respect to those collected on sample GGPE${}_{0}$. A similar behavior was observed also for the intensities of the bands ascribed to the O-H stretching of the water molecules, as it can be seen in Figure 6c, where the sum of the intensities of the four Raman bands increases, as well as the number of the PE layers. These results demonstrate that PE layers behave as surfaces for enhancing Raman scattering. In particular, the intensities of the Raman signals increase as the number of PE layers increases.

**Figure 7:**
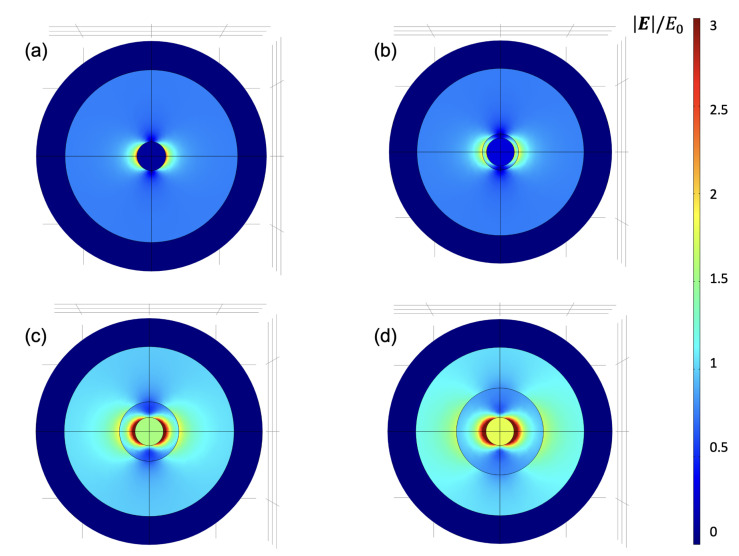
Finite element method (FEM) simulations reporting the local enhancement near-field around (**a**) GGPE${}_{0}$, (**b**) GGPE${}_{3}$, (**c**) GGPE${}_{11}$, and (**d**) GGPE${}_{21}$.

We can explain this behavior by considering two aspects: (i) By adding the PE layers, the plasmonic band of the GNPs shifts from 514 nm (GGPE${}_{0}$ sample) to 538 nm (GGPE${}_{11}$ and GGPE${}_{21}$ samples) by matching the position of the plasmonic peak with the wavelength used to perform the SERS analysis. In the case of GGPE${}_{0}$, the distance between the maximum of the plasmonic band that corresponds to the maximum enhancement of the electric near-field around the nanoparticles and the laser at 532 nm is of ∼14 nm; in the case of GGPE${}_{3}$, it is ∼10 nm, while in the case of GGPE${}_{11}$ and GGPE${}_{21}$, it is ∼6 nm. (ii) The second aspect that we consider is related to the increase in the intensity of the plasmonic band due to the presence of PE layers: The absorbance of GGPE${}_{0}$ at 514 nm is ∼0.07; for the GGPE${}_{21}$, it is ∼0.21, and the extra absorbance is due to the increase in the permittivity of the surrounding medium around GNPs. Finite Element Method (FEM) simulations have been conducted to numerically demonstrate this aspect by means of Comsol Multiphysics. By calculating the enhancement of the near-field (|E|/$E_{0}$) at 532 nm, it is possible to see how its maximum increases as a function of the number of PE layers in the region around the GNP. A field map of the |E|/$E_{0}$, where $E_{0}$ is the electric field intensity of the incident field, is reported in Figure 7. It is possible to distinguish that the spatial region affected by an enhancement of the near-field greater than 1 is more extensive in the case of GGPE${}_{11}$ and GGPE${}_{21}$ (green and yellow region in Figure 7c,d) As a result of the near field calculation, it is possible to observe that |E|/$E_{0}$ proceeds from a maximum value of about 3.5 for the case of GNP without the PE layer (GGPE${}_{0}$—Figure 7a) up to about 5.5 for the case with 21 layers of PE (GGPE${}_{21}$—Figure 7d). Similarly, the near field around the shell increases as it extends spatially over a wider region. An increase in the thickness of the PE layer leads to an accompanying increase in the GNPs-PE’s diameter as well as an overall increase in the size of the entire structure, thereby increasing the absorption cross-section. The near-field enhancement increases initially with the dielectric thickness due to the increased size of the shell and stronger coupling between the inner core and the PE layer, characterized by a higher refractive index with respect to air. This effect gradually decreases after a threshold thickness of the PE layer due to an increase in the scattering cross-section.

The choice to add 21 layers around the GNPs is not accidental: It represents a good compromise for achieving a strong enhancement of the near field due to a change in permittivity around GNPs, and this can be exploited for the SERS signal. At the same time, we can optimize this process by matching the plasmonic resonance with the laser used for the SERS effect. A higher number of PE layers represent an excessive capping on GNPs, which would make the phenomenon less efficient by heavily dampening the electromagnetic enhancements, which would remain confined in the PE layer. A control experiment has been conducted by considering the PE layer without the GNP’s monolayer and with a higher number of PE layers. No variations in the intensities of the Raman signals have been appreciated. The deposited PE layer represents an innovative layer for SERS analysis. We demonstrated that it can be considered as a good candidate to prevent the direct contact between GNPs and a probed molecule without affecting the electromagnetic enhancement of plasmonic mono-layers. This represents a crucial point for the investigation of biological molecules, in which a direct interaction with plasmonic nano-objects can lead to a modification of their structure. Furthermore, the monolayer fabrication protocol and the deposition of the PE layers are inexpensive, easy to obtain, with the possibility of obtaining very large substrates in a very short period of time.

## 4. Conclusions

In this study, samples made by a glass substrate, a layer of GNP, and different layers of PE (0, 3, 11, and 21) have been studied in order to analyze the effect of the number of PE layers on Raman scattering. For this aim, an acetic acid solution at 10% *v*/*v* was used as a test substance and was characterized both in a cuvette and on the top of a glass substrate, and the relative Raman modes have been assigned. Subsequently, the acetic acid solution has been deposited on four substrates, and then the Raman spectra have been collected. In order to make a quantitative evaluation of the observed effects, all Raman spectra collected on different substrates have been fitted. The trends of the ratios of the intensities of the Raman bands of the acetic acid solution (acetic acid and water molecules) on the band at 1098 cm${}^{-1}$ ascribed to the substrates seem to indicate that there is an SERS effect that is related to the number of the PE layers. Such an effect has been explained considering that PE layers upshift the frequency of the plasmonic band of gold nanoparticles up to the laser frequency, increasing, in this manner, the photon–plasmon interaction. In addition, PE layers increase the permittivity of the medium surrounding GNPs, resulting in an increase in the intensities of the SERS bands.

## Data Availability

The data that support the findings of this study are available within the article.
